# Smad4 deficiency in hepatocytes attenuates NAFLD progression via inhibition of lipogenesis and macrophage polarization

**DOI:** 10.1038/s41419-025-07376-8

**Published:** 2025-01-31

**Authors:** Wei Yang, Xuanxuan Yan, Rui Chen, Xin Xin, Shuang Ge, Yongxiang Zhao, Xinlong Yan, Jinhua Zhang

**Affiliations:** 1 The College of Life Science and Bioengineering, Beijing Jiaotong University, Beijing, China; 2 National Center for International Research of Bio-Targeting Theranostics, Guangxi Key Laboratory of Bio-Targetubg Theranostics, Collaborative Innovation Center for Targeting Tumor Diagnosis and Therapy, Guangxi Talent Highland of Bio-Targeting Theranostics, Guangxi Medical University, Nanning, China; 3 Faculty of Environmental and Life Sciences, Beijing University of Technology, Beijing, China

**Keywords:** Mechanisms of disease, Cytokines

## Abstract

Nonalcoholic fatty liver disease (NAFLD), a major cause of chronic liver disorders, has become a serious public health issue. Although the Smad4 signaling pathway has been implicated in the progression of NAFLD, the specific role of Smad4 in hepatocytes in NAFLD pathogenesis remains unclear. Hepatocyte-specific knockout Smad4 mice (Alb^Smad4−/−^) were first constructed using the Cre-Loxp recombinant system to establish a high-fat diet induced NAFLD model. The role of Smad4 in the occurrence and development of NAFLD was determined by monitoring the body weight of mice, detecting triglycerides and free fatty acids in serum and liver tissue homogenates, staining the tissue sections to observe the accumulation of liver fat, and RT-qPCR detecting the expression of genes related to lipogenesis, fatty acid intake, and fatty acid β oxidation. The molecular mechanism of Smad4 in hepatocytes affecting NAFLD was therefore investigated through combining in vitro and in vivo experiments. Smad4 deficiency in hepatocytes mitigated NAFLD progression and decreased inflammatory cell infiltration. Moreover, Smad4 deficiency inhibited CXCL1 secretion by suppressing the activation of the ASK1/P38/JNK signaling pathway. Furthermore, targeting CXCL1 using CXCR2 inhibitors diminished hepatocyte lipogenesis and inhibited the polarization of M1-type macrophages. Collectively, these results suggested that Smad4 plays a vital role in exacerbating NAFLD and may be a promising candidate for anti-NAFLD therapy.

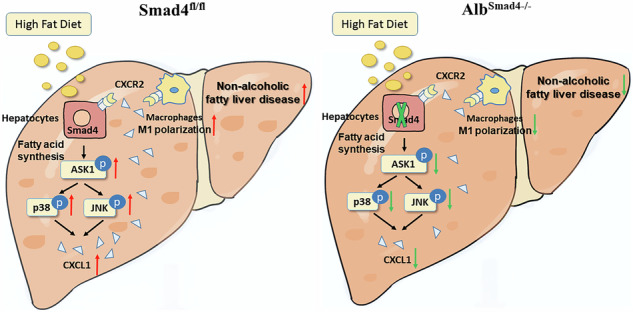

## Introduction

Nonalcoholic fatty liver disease (NAFLD) is characterized by the excessive accumulation of lipids within hepatocytes, due to factors other than alcohol consumption and other definitive liver damage sources [1]. NAFLD is a complex, multifactorial condition influenced by both environmental factors and genetic predispositions [2]. It encompasses a spectrum of pathological liver conditions of varying severity, ranging from isolated hepatic steatosis (NAFL) to steatohepatitis (NASH), which can further progress to liver fibrosis, cirrhosis, and hepatocellular carcinoma (HCC) [3]. With an estimated prevalence in nearly one-third of the global adult population, NAFLD has become a public health concern [4].

Hepatocytes, the main functional units of the liver, play a pivotal role in biotransformation, metabolism, and detoxification. During NAFLD progression, there is an excessive accumulation of triglyceride, free fatty acid (FFA), and cholesterol in hepatocytes, a process that is associated with insulin resistance that can lead to dysfunctional triglyceride synthesis and transport [5]. The excessive lipid accumulation induces lipotoxicity, which impairs mitochondrial function, triggers endoplasmic reticulum (ER) stress, and triggers inflammatory responses in hepatocytes due to reactive oxygen species (ROS)-induced inflammation [6, 7]. Concurrently, FFA accumulation in hepatocytes enhances mitochondrial β-oxidation, sensitizing the liver to oxidative stress and thereby exacerbating liver damage [8]. Moreover, lipotoxicity can disrupt the JNK pathway and Toll-like receptor cellular signaling pathways, thereby affecting hepatocyte metabolism [9, 10].

Liver macrophages comprise recruited monocyte-derived macrophages and resident Kupffer cells within the liver. Liver macrophages play a central role in the regulation of hepatocyte metabolism and maintenance of hepatic immunological tolerance [11, 12]. They can promote the progression of metabolic diseases by enhancing insulin resistance, hepatic steatosis, and oxidative stress in obese mice and rats [13, 14]. Under normal physiological conditions, liver macrophages exhibit a tendency toward the M2 phenotype, which suppresses inflammation by secreting interleukin (IL)-4 and IL-13 [15, 16]. However, during NAFLD progression, hepatic stellate cells (HSCs) activation triggers the secretion of pro-inflammatory cytokines, which increases the levels of lipopolysaccharide (LPS) in the blood. High levels of FFAs activate inflammasomes. These changes stimulate the onset of inflammation, with a concomitant increase in the proportion of M1 macrophages secreting pro-inflammatory cytokines [17–19].

The transforming growth factor beta (TGF-β) signaling pathway plays important roles in biological processes of cell growth, apoptosis, migration, and cancer development and progression [19]. Smad acts as a downstream signaling molecule in the TGF-β signaling pathway [20]. In mammals, eight different SMADs are further divided into three distinct classes: R-Smad (Smad1, 2, 3, 5, and 8), Co-Smad (Smad4), and I-Smad (Smad6 and 7) [21]. Smad4 is a central mediator of TGF-β signaling, which binds to nearly all Smad proteins regulated by activated receptors and helps regulate the expression of various downstream genes [22]. Hepatocyte Smad4 expression levels increase progressively as normal liver tissue progresses to NAFLD and finally to NASH [23, 24]. Smad4 deletion attenuates inflammation, fibrosis, and hepatocyte apoptosis in NASH [20]. After the administration of high-fat diet (HFD), Smad4 deletion in pancreatic β-cells improves blood glucose levels, insulin secretion, and glucose tolerance in obese mice [25]. Although other studies have investigated the role of Smad4 in liver disease, its specific molecular mechanism in hepatocytes in NAFLD remains unclear.

In this study, we examined the specific role of Smad4 in NAFLD progression using a mouse model with hepatocyte-specific Smad4 deletion. Our findings revealed that Smad4 deficiency in hepatocytes attenuates NAFLD development. Moreover, hepatocyte Smad4 was found to amplify CXCL1 secretion by facilitating the activation of the ASK1/P38/JNK signaling pathway. CXCL1, in turn, promotes hepatocyte lipogenesis and macrophage M1-type polarization via CXCR2 binding.

## Results

### Smad4 expression in hepatocytes is upregulated during NAFLD progression

**Fig. 1:**
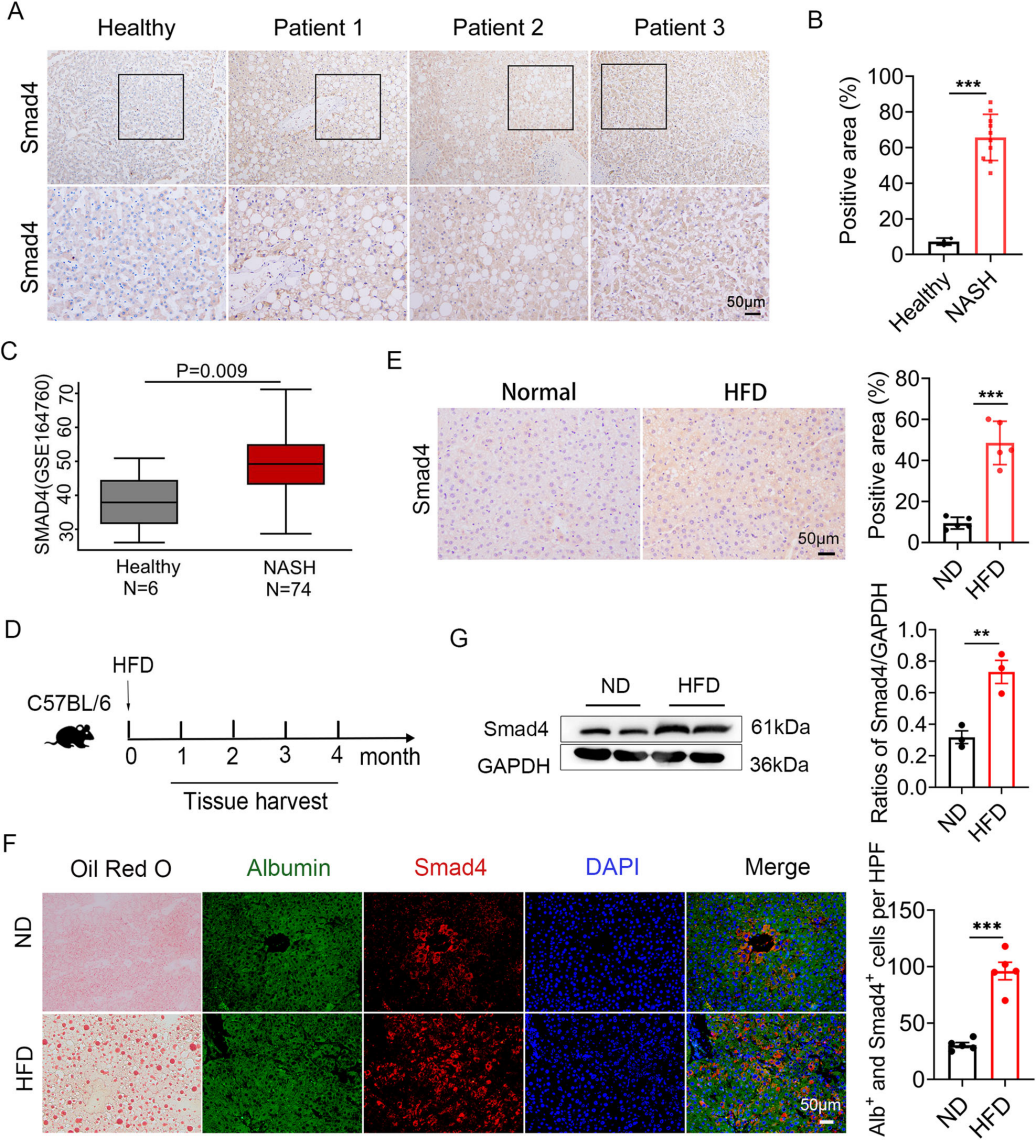
Smad4 expression in hepatocytes is upregulated during NAFLD progression. **A**, **B** Immunohistochemical staining of SMAD4 in a tissue microarray from NASH patients. **A** Representative Smad4 staining is shown Scale bar, 50 μm. **B** Statistical analysis ***P < 0.001. **C** Boxplots showing Smad4 expression levels in the GEO dataset GSE164760. Healthy: n = 6; NASH: n = 74. **D** Schematic illustration of HFD-induced mouse nonalcoholic fatty liver disease (NAFLD) model. **E** Representative staining of Smad4 in mouse liver samples and statistical analysis Scale bar, 50 μm ***P < 0.001. **F** Oil Red O staining, representative double staining, and statistical analysis of Albumin (green) and Smad4 (red) in mouse liver tissues. (HPF: High Power Field) Scale bar, 50 μm ***P < 0.001 . **G** The expression levels of Smad4 protein in mouse NAFLD liver tissues were determined using Western blot analysis. Smad4 was normalized to GAPDH **P < 0.01.

To elucidate the relationship between Smad4 expression and NASH, the expression of Smad4 in healthy liver and NASH tissues from patients was assessed by tissue microarray using immunohistochemistry. We observed that Smad4 expression was significantly upregulated in NASH tissues compared to that in healthy controls (Fig. 1A, B). Moreover, we analyzed the publicly available Gene Expression Omnibus dataset GSE164760 and compared Smad4 expression between healthy and NASH tissues. The analysis revealed a significant increase in Smad4 mRNA levels in the NASH group compared to healthy controls (Fig. 1C).

To corroborate these findings, we used a short-term HFD mouse model of NAFLD (Fig. 1D). Immunohistochemical staining demonstrated a significant upregulation of Smad4 expression in the hepatocytes of fatty liver tissues (Fig. 1E). Oil Red O staining revealed obvious vacuole of lipid droplets in the HFD group (Fig. 1F). Double immunofluorescence staining confirmed that Smad4 was expressed in most hepatocytes **(**Fig. 1F). Consistent with this, Western blot analysis indicated a significant increase in Smad4 protein levels in fatty liver tissues of HFD-treated mice (Fig. 1G).

Collectively, these results suggest that Smad4 is activated in hepatocytes during NAFLD, indicating a potentially crucial role for Smad4 in NAFLD pathogenesis.

### Hepatocyte-specific deletion of Smad4 attenuates high-fat diet-induced non-alcoholic fatty liver disease

**Fig. 2:**
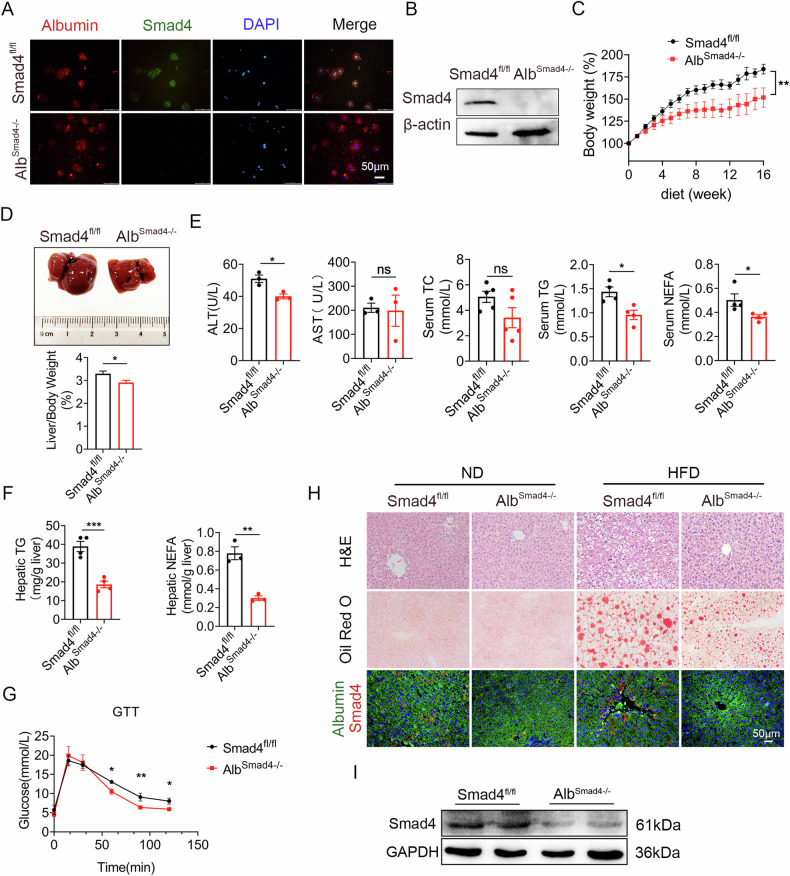
Hepatocyte-specific deletion of Smad4 attenuates high-fat diet-induced non-alcoholic fatty liver disease. Smad4^fl/fl^ and Alb^Smad4−/−^ mice were fed an HFD for 4 months to establish NAFLD model (n = 5 per group). Data are representative of at least three independent experiments. **A** Representative double staining of Albumin (red) and Smad4 (green) in primary hepatocytes. Scale bars, 50 μm. **B** The protein level of Smad4 in primary hepatocytes was determined by Western blot. **C** Body weight changes in Smad4^fl/fl^ and Alb^Smad4−/−^ mice were monitored during the NAFLD model construction **P < 0.01. **D** Representative photographs of the liver specimens and liver weight to body weight ratio *P < 0.05. **E** Serum alanine aminotransferase (ALT), aspartate aminotransferase (AST), total cholesterol (TC), triglycerides (TG), and non-esterified fatty acids (NEFA) levels *P < 0.05. **F** Hepatic TG and NEFA levels **P < 0.01 and ***P < 0.001. **G** Glucose tolerance test (GTT) in Smad4^fl/fl^ and Alb^Smad4−/−^ mice fed an HFD for 4 months *P < 0.05 and **P < 0.01. **H** Representative staining of hematoxylin and eosin (H&E), Oil Red O staining, and double staining of Albumin(red) and Smad4 (green) in liver tissues. Scale bar, 50 μm. **I** The protein levels of Smad4 in liver tissues were determined using Western blot analysis.

To further investigate the role of Smad4 in hepatocytes during NAFLD, we used a conditional Smad4 deletion approach in murine hepatocytes, as previously described [26]. We generated hepatocyte-specific Smad4 knockout mice (Albumin-cre; Smad4^flox/flox^, Alb^Smad4−/−^) by crossing mice carrying the Loxp-flanked Smad4 allele with Albumin-cre mice. The Alb^Smad4−/−^ mice were born at the expected Mendelian ratio, viable, and fertile. Smad4^fl/fl^ littermates were used as controls. Smad4 deletion in primary hepatocytes of Alb^Smad4−/−^ mice was confirmed using double immunofluorescence staining and Western blot (Fig. 2A, B). In addition, Smad4 was expressed in non-hepatocyte cells of Alb^Smad4−/−^ mice (Supplementary Fig. 1A).

To delineate the role of Smad4 in NAFLD, we established an HFD-induced NAFLD model using Alb^Smad4−/−^ mice and their control littermates. In response to HFD feeding, Alb^Smad4−/−^ mice demonstrated a less pronounced increase in both body and liver weight compared with control littermates (Fig. 2C, D). After 3 months of HFD, Alb^Smad4−/−^ mice displayed lower serum alanine aminotransferase (ALT), triglyceride (TG), and non-esterified fatty acid (NEFA) levels, while serum aspartate aminotransferase (AST) and total cholesterol (TC) levels were similar between Alb^Smad4−/−^ and control mice (Fig. 2E). Furthermore, hepatic TG and NEFA levels were notably reduced in Alb^Smad4−/−^ mice compared with Smad4^fl/fl^ mice (Fig. 2F). Alb^Smad4−/−^ mice also exhibited impaired glucose tolerance compared with Smad4^fl/fl^ mice (Fig. 2G). The absence of Smad4 in hepatocytes in Alb^Smad4−/−^ mice was further validated using double immunofluorescence staining and Western blot (Fig. 2H, I). Deletion of Smad4 in hepatocytes resulted in decreased fat accumulation, as evidenced by hematoxylin and eosin and Oil Red O staining. No significant differences were observed between Alb^Smad4−/−^ and Smad4^fl/fl^ mice who were fed a normal diet (Fig. 2H). Collectively, these data suggest that hepatocyte-specific Smad4 deficiency attenuates the development of HFD-induced NAFLD.

### Smad4 deficiency in hepatocytes attenuated liver inflammation and CXCL1 secretion

**Fig. 3:**
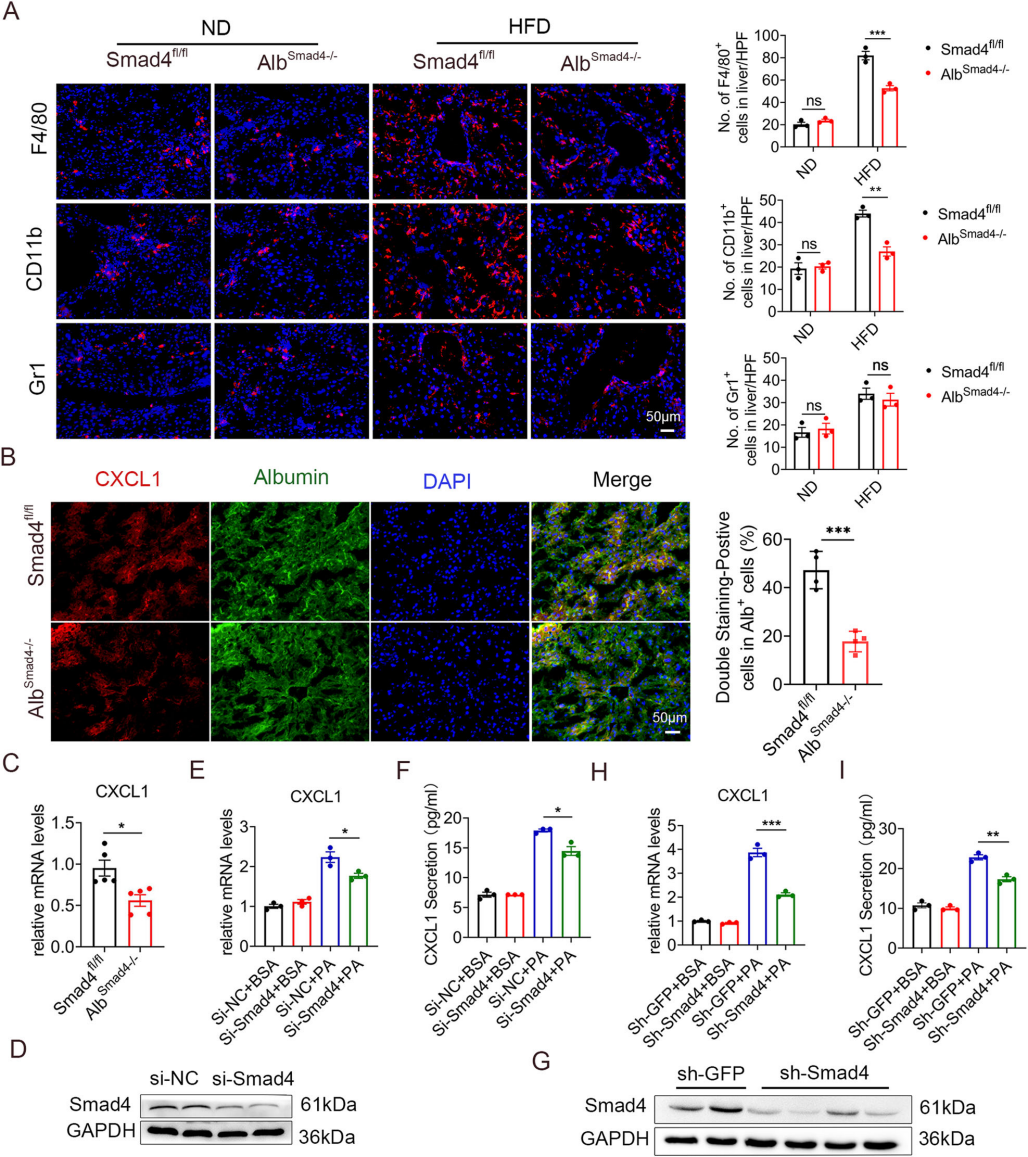
Smad4 deficiency in hepatocytes attenuated liver inflammation and CXCL1 secretion. Smad4^fl/fl^ and Alb^Smad4−/−^ mice were fed an HFD for 4 months to establish the NAFLD model (n = 5 per group). Data are representative of at least three independent experiments. **A** Immunofluorescence detection and statistical analysis of F4/80, CD11b, and Gr1 expression in NAFLD liver tissues. Scale bar, 50 µm **P < 0.01; ***P < 0.001. **B** Double immunofluorescence staining for CXCL1 (red) and Albumin (green) in liver tissue. Scale bar, 50 μm ***P < 0.001. **C** The relative mRNA expression of CXCL1 in liver tissues was measured using RT-qPCR, *P < 0.05. **D**–**I** AML12 cells were transfected with si-NC or si-Smad4, respectively, cultured with BSA or palmitic acid (PA, 500 μM) for 24 h. **D** The protein levels of Smad4 in control si-NC and si-Smad4 AML12 cells were determined using Western blot. **E** Relative mRNA expression of CXCL1 was measured using RT-qPCR in AML12 cells *P < 0.05. **F** ELISA verification of culture supernatants from AML12 cells *P < 0.05. **G**–**I** AML12 cells transfected with lentiviral vectors for control or Smad4 knockdown were then cultured with BSA or PA (500 μM) for 24 h. **G** Western blot analysis of Smad4 protein expression in sh-GFP and sh-Smad4 AML12 cells. **H** The mRNA levels of CXCL1 were measured by RT-qPCR in AML12 cells ***P < 0.001. **I** Secretory protein levels of CXCL1 in AML12 cell medium were determined using ELISA. **P < 0.01.

To investigate whether Smad4 modulates liver inflammatory cell infiltration and hepatocyte proliferation, we stained liver tissues of Alb^Smad4−/−^ and Smad4^fl/fl^ mice via immunofluorescence. The infiltration of F4/80^+^ macrophages and CD11b^+^ monocytes was diminished in Alb^Smad4−/−^ mice compared with that in Smad4^fl/fl^ mice when fed an HFD. When fed the normal diet, immune cell infiltration was similar between Alb^Smad4−/−^ and control mice (Fig. 3A). During NAFLD progression, activated chemokines evoke multiple cellular and tissue responses, including hepatocyte proliferation, activation, necrosis, angiogenesis, and immune cell recruitment [27, 28]. Notably, CXCL1 is a key gene involved in NAFLD progression. Therefore, we examined CXCL1 expression in the livers of Alb^Smad4−/−^ and Smad4^fl/fl^ mice through double immunofluorescence staining. CXCL1 levels were significantly lower in the livers of Alb^Smad4−/−^ mice than those in Smad4^fl/fl^ mice (Fig. 3B). This decrease in CXCL1 levels was further confirmed using quantitative reverse transcription polymerase chain reaction (RT-qPCR) in hepatocytes of NAFLD of Alb^Smad4−/−^ mice (Fig. 3C).

To further clarify the function of Smad4 in hepatocytes, we knocked down Smad4 in AML12 cells using siRNA and verified Smad4 protein levels using Western blot (Fig. 3D). The cells were then treated with palmitic acid (PA) for 24 h to simulate an in vitro NAFLD environment. RT-qPCR analysis revealed that Smad4 deficiency mitigated the PA-induced CXCL1 expression in hepatocytes (Fig. 3E). The protein expression level of CXCL1 was further analyzed using enzyme-linked immunosorbent assay (ELISA) with AML12-conditioned medium (CM). Smad4 deficiency partly reduced the secretion of CXCL1 in PA-induced AML12 CM (Fig. 3F).

In addition, AML12 cells were transfected with a lentiviral vector to knock down Smad4, and the expression levels of Smad4 protein were measured using Western blot (Fig. 3G). In line with previous results, both the expression and secretion of CXCL1 in PA-stimulated Smad4-knockdown AML12 cells were significantly reduced compared to those in control cells (Fig. 3H, I). Taken together, these results suggest that hepatocyte-specific deletion of Smad4 lessens liver inflammation and CXCL1 secretion.

### Hepatocyte Smad4 promotes CXCL1 secretion via the ASK1-P38-JNK signaling pathway

**Fig. 4:**
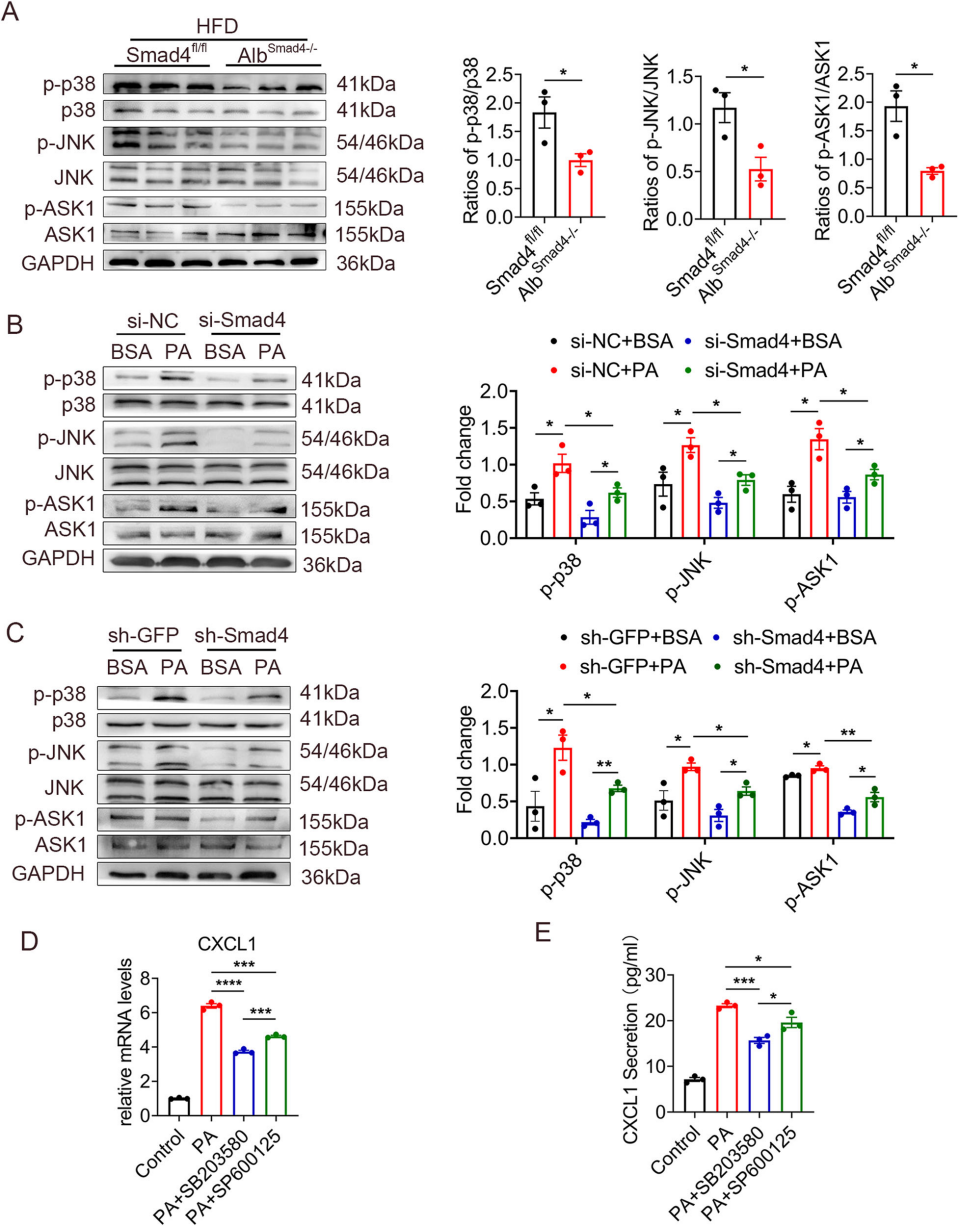
Hepatocyte Smad4 promotes CXCL1 secretion via the ASK1-P38-JNK signaling pathway. Groups of Smad4^fl/fl^ and Alb^Smad4−/−^ mice were fed an HFD for 4 months to establish the NAFLD model (n = 5 per group). **A** The expression levels of p38, p-p38, JNK, p-JNK, ASK1, and p-ASK1 in the liver were measured using Western blot analysis. Protein densities were quantified using densitometry. Phospho-protein levels were normalized to the total protein levels *P < 0.05. **B** The expression levels of p38, p-p38, JNK, p-JNK, ASK1, and p-ASK1 proteins were measured using Western blot analysis in si-NC and si-Smad4 AML12 cells treated with PA (500 μM) for 24 h. Phospho-proteins were normalized to total proteins *P < 0.05. **C** The expression levels of p38, p-p38, JNK, p-JNK, ASK1, and p-ASK1 proteins were measured using Western blot analysis in sh-GFP and sh-Smad4 AML12 cells treated with PA (500 μM) for 24 h. Phospho-protein levels were normalized to total protein levels *P < 0.05 and **P < 0.01. **D**, **E** After pretreatment of AML12 cells with SB203580 (P38 MAPK inhibitor) or SP600125 (JNK inhibitor) for 2 h, 500 μM PA was co-incubated for 24 h. **D** The mRNA levels of CXCL1 were measured using RT-qPCR in AML12 cells ***P < 0.001. **E** Secretory protein levels of CXCL1 were measured using ELISA in AML12 cells *P < 0.05 and ***P < 0.001.

Previous studies have highlighted the involvement of the JNK and p38 MAPK cascades in the regulation of CXCL1 secretion [29]. Accordingly, we examined the levels of total and phosphorylated proteins involved in ASK1, P38, and JNK signaling. We found that the expression of phosphorylated ASK1 (p-ASK1), p38 (p-p38), and JNK (p-JNK) was diminished in the liver tissue of HFD-treated Alb^Smad4−/−^ mice compared with Smad4^fl/fl^ mice (Fig. 4A).

In subsequent experiments, we used si-Smad4 and sh-Smad4 to knock down Smad4 in AML12 cells, which were then exposed to PA. The Western blot analysis showed that the ASK1, P38, and JNK signaling pathways were activated in hepatocytes in response to PA administration. However, this activation was remarkably suppressed by Smad4 knockdown, suggesting that Smad4 knockdown considerably inhibited PA-induced activation of the ASK1–P38–JNK pathway in vitro (Fig. 4B, C).

To further elucidate the signaling pathways involved in the induction of CXCL1 secretion by Smad4, we employed the JNK inhibitor (SP600125) and the p38 MAPK inhibitor (SB203580). Both inhibitors suppressed PA-induced CXCL1 secretion and mRNA expression, as evidenced using RT–qPCR and ELISA analysis (Fig. 4D, E). In conclusion, these results suggest that Smad4 facilitates CXCL1 secretion via the ASK1-P38-JNK signaling pathways during NAFLD progression.

### CXCL1 promotes fatty acid synthesis in hepatocytes by binding to CXCR2

**Fig. 5:**
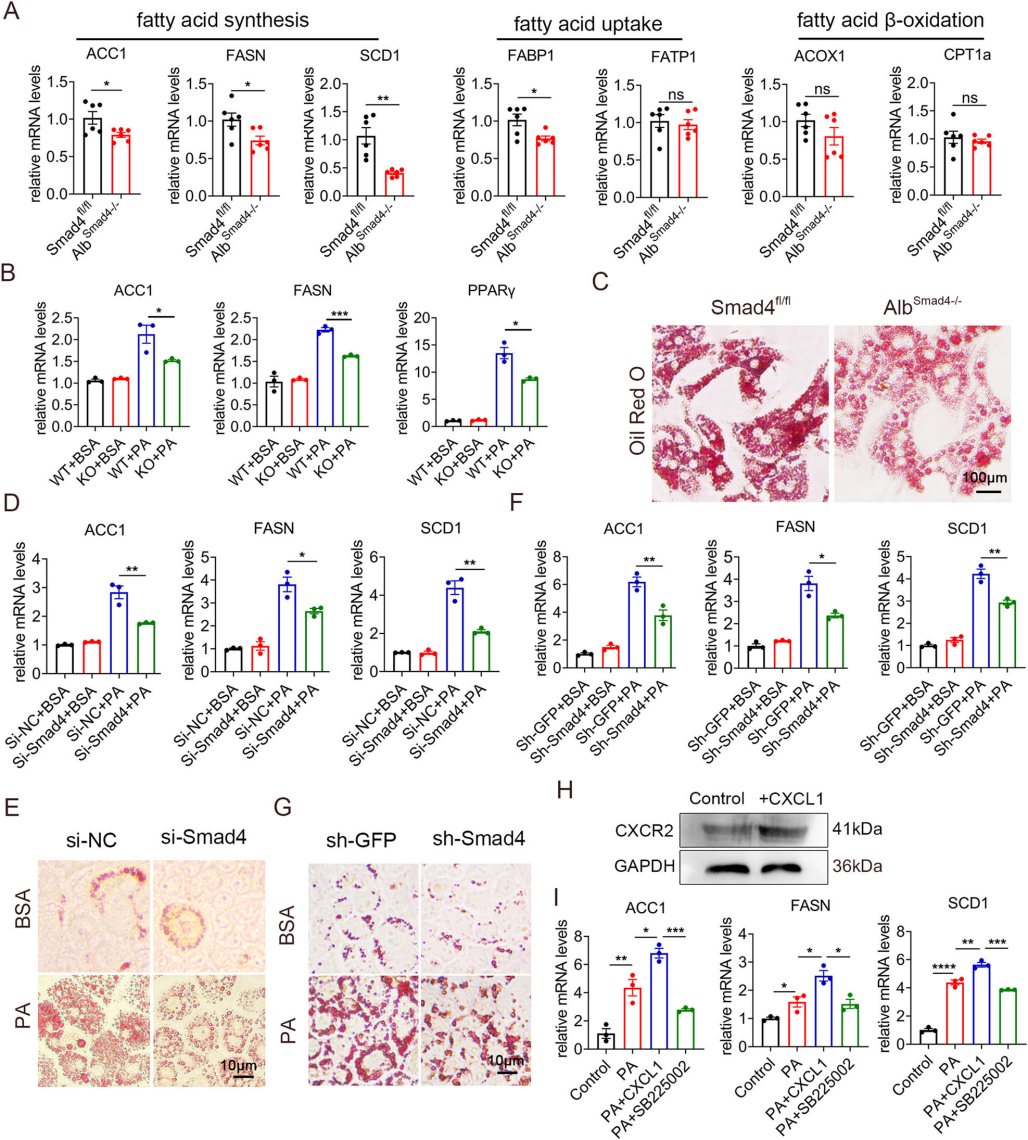
CXCL1 promotes fatty acid synthesis in hepatocytes by binding to CXCR2. Groups of Smad4^fl/fl^ and Alb^Smad4−/−^ mice were fed an HFD for 4 months to establish the NAFLD model (n = 5 per group). **A** The mRNA levels of ACC1, FASN, SCD1, FABP1, FATP1, ACOX1, and CPT1a in NAFLD liver tissues were measured using RT-qPCR analysis *P < 0.05 and **P < 0.01. **B**–**G** Primary hepatocytes and AML12 cells were treated with BSA or PA (500 μM) for 24 h. **B** The mRNA levels of ACC1, FASN, and PPARγ in primary hepatocytes were determined using RT-qPCR analysis *P < 0.05 and ***P < 0.001. **C** Oil Red O staining of primary hepatocytes. Scale bar, 100 μm. **D** The mRNA levels of ACC1, FASN, and SCD1 were determined using RT-qPCR analysis in AML12 cells with si-NC or si-Smad4 transfection *P < 0.05 and **P < 0.01. **E** Oil Red O staining of AML12 cells transfected with si-NC or si-Smad4. Scale bar, 10 μm. **F** The mRNA levels of ACC1, FASN, and SCD1 were determined using RT-qPCR analysis in AML12 cells transfected with sh-GFP or sh-Smad4 *P < 0.05 and **P < 0.01. **G** Oil Red O staining of AML12 cells transfected with sh-GFP or sh-Smad4, respectively. Scale bar, 10 μm. **H** The protein levels of CXCR2 in AML12 cells treated with 50 ng/mL CXCL1 were detected by Western blot. **I** AML12 cells were cultured with 500 μM PA, 50 ng/mL CXCL1 recombinant protein, and 500 nM SB225002 (inhibitor of CXCL1 receptor CXCR2) for 24 h. The mRNA levels of ACC1, FASN, and SCD1 were determined using RT-qPCR analysis in AML12 cells *P < 0.05, **P < 0.01 and ***P < 0.001.

To further investigate the role of Smad4 in hepatic lipid deposition, RT-qPCR was used to determine the expression levels of genes involved in fatty acid synthesis and consumption. We found that Smad4 deficiency considerably inhibited the expression of genes critical for fatty acid synthesis (ACC1, FASN, and SCD1) and fatty acid binding protein 1 (FABP1). However, we observed no substantial differences in the expression of genes related to fatty acid uptake (FATP1) and fatty acid β-oxidation (CPT1a and ACOX1) (Fig. 5A).

To corroborate these findings, we isolated primary hepatocytes from Alb^Smad4−/−^ and Smad4^fl/fl^ mice and then exposed them to PA. Following Smad4 knockout, the expression of genes responsible for fatty acid synthesis (ACC1, FASN, and PPAR-γ) was substantially reduced in primary hepatocytes (Fig. 5B). We further observed decreased lipid deposition in primary hepatocytes of Alb^Smad4−/−^ mice compared with that in Smad4^fl/fl^ mice following PA treatment, as evidenced by Oil Red O staining **(**Fig. 5C**)**. This conclusion was confirmed using si-Smad4 AML12 and sh-Smad4 AML12 cells (Fig. 5D–G). Taken together, these findings suggest that hepatocyte Smad4 promotes NAFLD development primarily through fatty acid synthesis.

CXCL1 binds to specific receptor CXCR2. We then examined whether CXCL1-induced fatty acid synthesis occured in hepatocytes. We assessed the expression levels of CXCR2 in hepatocytes using Western blot (Fig. 5H). To further elucidate the molecular mechanisms underlying the effects of CXCL1 on fatty acid synthesis, we cultured AML12 cells with CXCL1 recombinant protein and analyzed the expressions of genes related to fatty acid synthesis using RT-qPCR. We observed that ACC1, FASN, and SCD1 were substantially upregulated by CXCL1. However, when CXCR2 activation was inhibited by the CXCR2 inhibitor, SB225002, in AML12 cells, CXCL1-induced ACC1, FASN, and SCD1 expression was reduced (Fig. 5I). These results suggest that CXCL1 induces fatty acid synthesis in hepatocytes via CXCR2.

### CXCL1 promotes macrophage M1 polarization

**Fig. 6:**
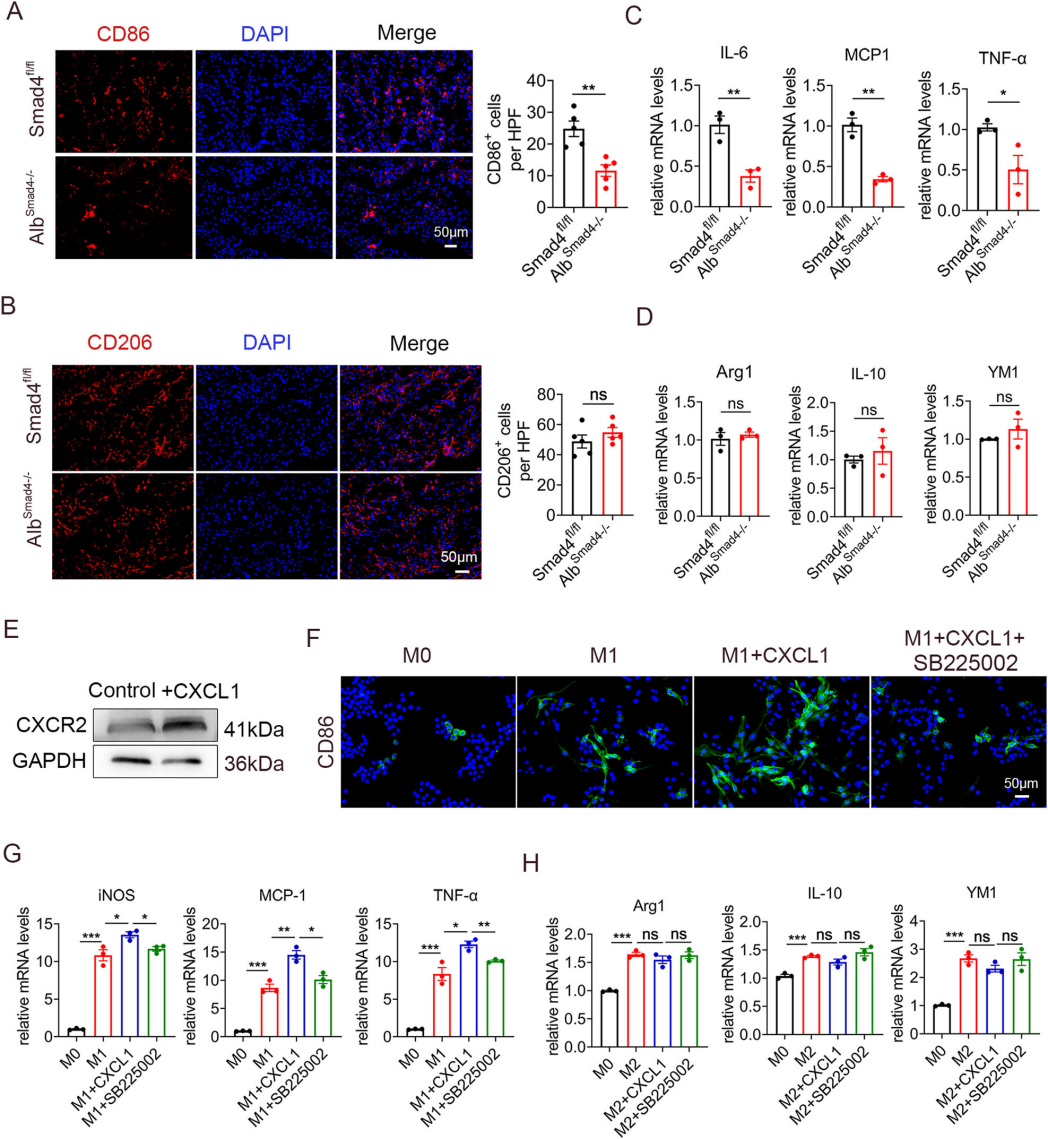
CXCL1 promotes macrophage M1 polarization. Smad4^fl/fl^ and Alb^Smad4−/−^ mice were fed an HFD for 4 months to establish the NAFLD model (n = 5 per group). Data are representative of at least three independent experiments. **A**, **B** Representative staining and statistical analysis of CD86 and CD206 expression in liver tissues Scale bar, 50 μm. **C**, **D** The mRNA levels of IL-6, MCP1, TNF-α, Arg1, IL-10, and YM1 in liver tissues were determined using RT-qPCR analysis *P < 0.05 and **P < 0.01. **E** The protein levels of CXCR2 in Raw264.7 cells treated with 50 ng/mL CXCL1 were detected by Western blot. **F**, **G** Raw264.7 cells were cultured with 100 ng/mL LPS, 10 ng/mL IFN-γ, 50 ng/mL CXCL1 recombinant protein, and 500 nM SB225002 for 24 h. **F** Immunofluorescence staining of CD86 in RAW264.7 cells Scale bar, 50 μm. **G** The mRNA levels of iNOS, MCP1, and TNF-α in RAW264.7 cells were determined using RT-qPCR analysis *P < 0.05, **P < 0.01 and ***P < 0.001. **H** Raw264.7 cells were cultured with 20 ng/mL IL-4, 20 ng/mL IL-13, 50 ng/mL CXCL1 recombinant protein, and 500 nM SB225002 for 48 h. The mRNA levels of Arg1, IL-10, and YM1 in RAW264.7 cells were determined using RT-qPCR analysis ***P < 0.001.

Aberrant lipid-mediated hepatic inflammatory-immune dysfunction and chronic low-grade inflammation play important roles in NAFLD pathogenesis. Macrophage polarization is an important mechanism that regulates inflammatory responses [30]. Therefore, we assessed the quantities of CD86^+^ (M1 marker) and CD206^+^ (M2 marker) macrophages in NAFLD tissues. Our findings revealed a substantial decrease in M1 macrophages in Alb^Smad4−/−^ mice compared with Smad4^fl/fl^ mice, while M2-like macrophages remained comparable (Fig. 6A, B). We further evaluated the expression levels of genes associated with M1-like (IL-6, MCP1, and TNF-α) and M2-like (Arg1, IL-10, and YM1) phenotypes using RT-qPCR and found similar results (Fig. 6C, D). Given that the absence of hepatocyte Smad4 resulted in diminished CXCL1 secretion, we postulated that hepatocyte Smad4 might facilitate macrophage M1 polarization via CXCL1. We confirmed the expression of CXCR2 in macrophages using western blot (Fig. 6E). We cultured RAW264.7 cells with LPS and IFN-γ for 24 h to induce M1 polarization. It was found that LPS and IFN-γ activated the expression of CD86 in macrophages in comparison with the control group, CXCL1 recombinant proteins further upregulated CD86. This expression was further augmented by the addition of CXCL1 recombinant proteins, as evidenced by immunofluorescence staining. However, the treatment of RAW264.7 cells with the CXCR2 inhibitor SB225002 eliminated CXCL1-induced expression of CD86 (Fig. 6F). Following M1 polarization induction, the expression levels of iNOS, MCP1, and TNF-α were significantly increased in RAW264.7 cells compared with those in the control group, and the addition of exogenous CXCL1 recombinant protein further amplified M1-related gene expression (Fig. 6G).

For M2 polarization, RAW264.7 cells were cultured with IL-4 and IL-13 for 48 h. This treatment activated the expression of Arg1, IL-10, and YM1 in macrophages compared with the control group. No significant differences were observed after the administration of CXCL1 and SB225002 (Fig. 6H). These findings suggest that CXCL1 promotes the M1-type polarization of macrophages via CXCR2.

## Discussion

Smad4 is a general mediator of the TGF-β and bone morphogenetic protein (BMP) signaling pathways, which significantly contribute to intracellular signal transduction and a myriad of cellular processes [19]. However, owing to its ubiquitous expression, the specific role and molecular mechanism of Smad4-mediated signaling in NAFLD progression remain elusive. Our study illustrated that hepatocyte-specific Smad4 expression promoted NAFLD development by enhancing CXCL1 secretion. Targeted deficiency of hepatocyte-specific Smad4 signaling curbed the progression of NAFLD in HFD-fed mice. Hepatocyte-specific genetic deficiency of Smad4 inhibited fatty acid synthesis and macrophage M1 polarization. Moreover, Smad4 in hepatocytes accelerated CXCL1 secretion to enhance fatty acid synthesis and macrophage M1 polarization by activating the ASK-P38-JNK signaling pathway.

NAFLD is now recognized as a steatotic liver disease closely associated with metabolic syndrome. The relationship between NAFLD and liver inflammation has been extensively studied. Several studies indicate the pivotal role of TGF-β/Smad signaling in metabolic syndrome and related disorders [31, 32]. Our results suggested that Smad4 is activated in hepatocytes during NAFLD **(**Fig. 1). We found that there was no difference in Smad4 nucleus expression between ND and HFD groups detected by Western blot in vivo (Supplementary Fig. 2A). Similar results were obtained by using AML12 cells. In addition, the stimulation of TGF-β didn’t change Smad4 expression **(**Supplementary Fig. 2B**)**. Therefore, Smad4 activation didn’t depend on TGF-β **(**Supplementary Fig. 2A, B**)**. Our previous study demonstrated that the targeted knockout of Smad4 in hepatocytes attenuates hepatic inflammatory cell infiltration and fibrosis during the progression of CCl_4_ induced liver fibrosis [26]. Disruption of the Smad4 pathway alleviated spontaneous liver injury, hepatic inflammatory cell infiltration, fibrosis, and HCC induced by TAK1 deletion in hepatocytes [33]. Kundu et al. confirmed that the SIRT4/SMAD4 axis played a vital role in HFD-fed induced liver fibrosis. Upregulation of SIRT4 and downregulation of Smad4 can potentially counteract lipid accumulation, inflammation, and fibrosis during NAFLD progression [34]. Hepatocyte-specific deletion of Smad4 markedly reduced the expression of fibrosis, hepatocyte apoptosis-, and inflammation-related genes during NASH progression [20]. Collectively, our results support this conclusion and demonstrate that hepatocyte-specific Smad4 deficiency alleviates HFD-fed induced NAFLD.

Hepatocytes comprise the largest number of parenchymal cells in the liver and are the primary undertakers of liver function. With little or no alcohol intake, steatosis in more than 5% of hepatocytes is diagnosed as NAFLD [35]. Increased lipid influx into the liver or reduced lipid disposal precipitates hepatic steatosis, primarily instigated by a HFD, genetic predisposition, gut microbiota, and upregulated expression of lipid transcription factors (e.g, SREBP1c, chREBP, and PPAR-γ) [36]. Abnormal accumulation of lipotoxic lipids, including fatty acids, diacylglycerols, and cholesterol in the liver, induces hepatocellular injury, including lipotoxicity, mitochondrial dysfunction, oxidative stress, ER stress, and severe inflammatory responses [37]. Our results revealed that Smad4 deficiency in hepatocytes curtailed the secretion of CXCL1, which consequently mitigated hepatocyte fatty acid synthesis and macrophage M1 polarization.

A recent integrative analysis of mild and severe NAFLD identified CXCL1 as one of the five hub genes. In vitro and in vivo experiments demonstrated that high-fat conditions increased CXCL1 levels in both the liver tissue and hepatocytes, which correlated with the duration of HFD feeding and PA concentration, consistent with our findings [28]. CXCL1 is an important chemokine that is implicated in the progression of numerous inflammatory diseases [38, 39]. In the liver, CXCL1 is predominantly expressed in hepatocytes, with lower level expression in HSCs and liver-sinusoidal endothelial cells [40]. CXCL1 primarily binds to the receptor CXCR2 and recruits neutrophils to inflammation sites. Previous studies have shown that CXCL1 chemokines are induced and released by the P38 MAPK and JNK signaling pathways in human pulmonary epithelial cells and vascular endothelial cells [29, 41]. In this study, we demonstrated that hepatocyte Smad4 expression stimulated CXCL1 secretion via the ASK1, P38 MAPK, and JNK signaling pathways, thereby promoting the progression of NAFLD. However, the potential role of CXCL1 on hepatocytes via other pathways warrants further investigation.

M1 macrophages are key players in chronic inflammatory diseases, such as atherosclerosis, rheumatoid arthritis (RA), and inflammatory bowel disease (IBD) [42–44]. Macrophages play a significant role in NAFLD pathogenesis, as evidenced by the prevention of inflammatory cell recruitment, hepatic steatosis, and hepatic insulin resistance in Kupffer cell -depleted mice [13, 45]. In the NAFLD environment, macrophages are regulated by various molecular signals to polarize toward the M1 phenotype [46]. Cytokines secreted by M1 liver macrophages are also likely to repress fatty acid oxidation and potentiate triglyceride synthesis [47, 48]. In line with this, we demonstrated that Smad4 deficiency in hepatocytes inhibits the transition of macrophages to the M1 phenotype in an NAFLD model. Interestingly, the secretion of CXCL1 does not affect macrophage M2 polarization. CXCL1 has been reported to play a crucial role in M1 macrophage polarization during cerebral aneurysm development [49]. Consistent with this, our study showed that hepatocyte Smad4 promoted macrophage M1 polarization by facilitating CXCL1 secretion. Whether Smad4 influences NAFLD via other molecular mechanisms remains unclear.

In conclusion, our study revealed that Smad4 expression in hepatocytes plays a crucial role in the development of NAFLD. Smad4 in hepatocytes amplified lipid accumulation and M1 macrophage polarization by stimulating CXCL1 secretion, thereby promoting NAFLD progression. Smad4 in hepatocytes may represent a potential preventive and therapeutic target for NAFLD.

## Materials and Methods

Some detailed information was provided in supplementary data. The details of RT-qPCR primers are described in supplementary material, Table S1.

### Mice

Smad4^flox/flox^ and Alb^Smad4−/−^ mice on a C57BL/6 background have been described previously [26, 50]. Mice with a conditional knockout of Smad4 in hepatocytes expressing Albumin (Alb^Smad4−/−^) were generated by crossing Alb-Cre and Smad4^flox/flox^ mice. The mice in the control group are cre-negative littermates. All mice were maintained in specific pathogen-free and humidity- and temperature-controlled microisolator cages with a 12-h light/dark cycle at the Institute of Biophysics, Chinese Academy of Sciences. Alb^Smad4−/−^ mice and their littermate control mice which are used for the experiments were 5–6 weeks old.

### HFD-induced NAFLD model

The NAFLD model was administered in mice by feeding an HFD (60% of total energy from fat, Huafukang, Beijing, CN) continuously for 16 weeks. Mice that were administered a normal chow diet (ND, 10% of total energy from fat, Huafukang, Beijing, CN) served as controls.

### Cell lines and treatment

The AML12 and RAW264.7 cell lines were purchased from the American Type Culture Collection (ATCC; Manassas, VA, USA). The AML12 cell was cultured in DMEM/F12 medium (Gibco, Grand Island US) supplemented with 10% fetal bovine serum (FBS, PAN biotech, Adenbach, Germany), 1% penicillin/ streptomycin, 40 ng/mL dexamethasone (Solarbio, Beijing, China), and 1% insulin-transferrin-selenium (ITS, Procell, Wuhan, China). The RAW264.7 cell was cultured in DMEM/ 1640 supplemented with 10% FBS and 1% penicillin/ streptomycin. The cells were cultured at 37 °C with 5% CO_2_. AML12 cells were exposed to palmitic acid (500 μM) (Sigma, USA) for 24 h. AML12 cells were treated with inhibitor of P38 MAPK SB203580 (10 μM) (MedChemExpress, Princeton, NJ, USA) and inhibitor of JNK for SP600125 (10 μM) (MedChemExpress, Princeton, NJ, USA) for 2 h in advance. After incubation, the AML12 cells were challenged with 50 ng/mL CXCL1 recombinant protein (Sino Biological, Beijing, China) for 24 h for further analysis. AML12 and RAW264.7 cells were stimulated with 50 ng/mL CXCL1 recombinant protein and 500 nM inhibitor of CXCL1 receptor CXCR2 SB225002 (MedChemExpress, Princeton, NJ, USA) for 24 h for further analysis.

### Statistical analysis

All data were expressed as the mean ± SEM and analyzed using GraphPad Prism V8.0.2 software. Significant differences between mean values were obtained from three independent experiments. Differences between the two groups were compared using two-tailed unpaired Student’s t-test analysis. Two-way ANOVA was used for multiple comparisons. P < 0.05 was considered statistically significant.

## Supplementary information

Table S1

Original Western blot

Supplemental material

## Data Availability

All data generated or analyzed during this study are included in this article and its online supplementary material. Further inquiries can be directed to the corresponding author.
