# Supplementary material for: Smad4 deficiency in hepatocytes attenuates NAFLD progression via inhibition of lipogenesis and macrophage polarization
Source: Cell Death Dis. 2025 Jan 31;16(1):58. doi: 10.1038/s41419-025-07376-8 (PMC11785999; doi:10.1038/s41419-025-07376-8)
Supplement: Supplementary file 1 — Table S1 [file 41419_2025_7376_MOESM1_ESM.docx]

**Table S1. Real-time PCR primer sequences.**

| **Gene** | **Species** | **Primer sequence (5'–3')** |
| --- | --- | --- |
| *CXCL1* | Mouse | F: CCCTGAAGCTCCCTTGGTTC |
|  |  | R: TGGGGACACCTTTTAGCATCT |
| *ACC1* | Mouse | F: GTTCTGTTGGACAACGCCTTCAC |
|  |  | R: GGAGTCACAGAAGCAGCCCATT |
| *FANS* | Mouse | F: ACCTCCAGTCGTGAAACCAT |
|  |  | R: CTCAGCTGTGTCTTGGATGC |
| *SCD1* | Mouse | F: GCAAGCTCTACACCTGCCTCTT |
|  |  | R: CGTGCCTTGTAAGTTCTGTGGC |
| *PPARγ* | Mouse | F: TAGGTGTGATCTTAACTGCCG |
|  |  | R: GCATCGTGTAGATGATCTCAGGCA |
| *FABP1* | Mouse | F: AGGGGGTGTCAGAAATCGTG |
|  |  | R: CCCCCAGGGTGAACTCATTG |
| *FATP1* | Mouse | F: TGAGAGTCTCGTTGGGTTGC |
|  |  | R: CAAAAGCTCTCAGCCCCTCA |
| *Acox1* | Mouse | F: GGAACCTGTTGGCCTCAATTA |
|  |  | R: CAAAGGCTCAGGATGCCCTC |
| *CPT1a* | Mouse | F: GGACTCCGCTCGCTCATTC |
|  |  | R: GGCAGATCTGTTTGAGGGCT |
| *IL-6* | Mouse | F: TACCACTTCACAAGTCGGAGGC |
|  |  | R: CTGCAAGTGCATCATCGTTGTTC |
| *MCP1* | Mouse | F: GCTACAAGAGGATCACCAGCAG |
|  |  | R: GTCTGGACCCATTCCTTCTTGG |
| *TNF-α* | Mouse | F: TGAGGTCAATCTGCCCAAGT |
|  |  | R: GGGGTCAGAGTAAAGGGGTC |
| *Arg1* | Mouse | F: CTCCAAGCCAAAGTCCTTAGAG |
|  |  | R: AGGAGCTGTCATTAGGGACATC |
| *YM1* | Mouse | F: CTCAACCTGGACTGGCAGTA |
|  |  | R: CTGCTCCTGTGGAAGTGAGT |
| *IL-10* | Mouse | F: GCTCTTACTGACTGGCATGAG |
|  |  | R: CGCAGCTCTAGGAGCATGTG |
| *iNOS* | Mouse | F: CGGAGATCAATGTGGCTGTG |
|  |  | R: GAAGGACTCTGAGGCTGTGT |
| *GAPDH* | Mouse | F: CATCACTGCCACCCAGAAGACTG |
|  |  | R: ATGCCAGTGAGCTTCCCGTTCAG |
