# Supplementary material for: Smad4 deficiency in hepatocytes attenuates NAFLD progression via inhibition of lipogenesis and macrophage polarization
Source: Cell Death Dis. 2025 Jan 31;16(1):58. doi: 10.1038/s41419-025-07376-8 (PMC11785999; doi:10.1038/s41419-025-07376-8)
Supplement: Supplementary file 2 — Original Western blot [file 41419_2025_7376_MOESM2_ESM.pdf]

Original Western blot

Fig 1

1-G

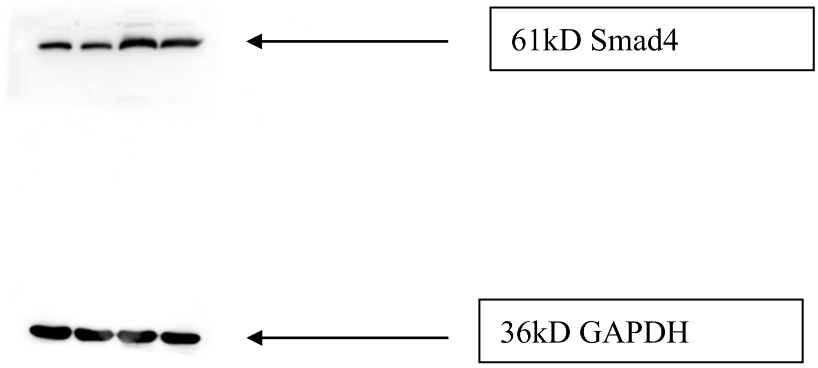

Fig2

2-B

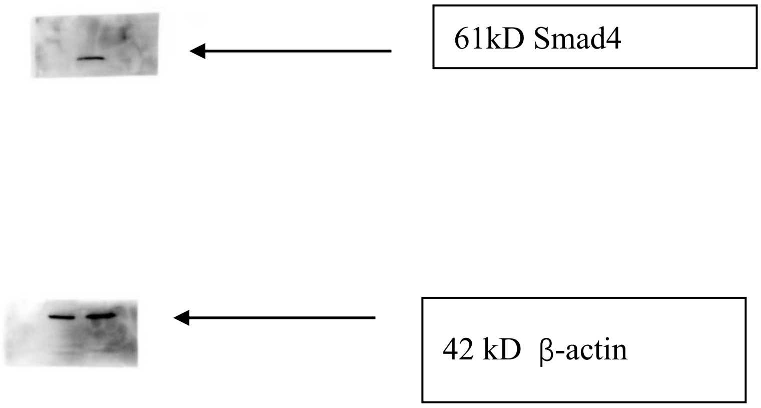

2-I

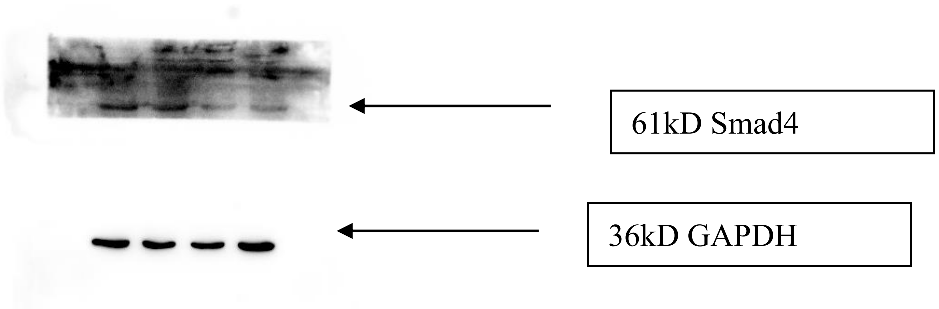

总:

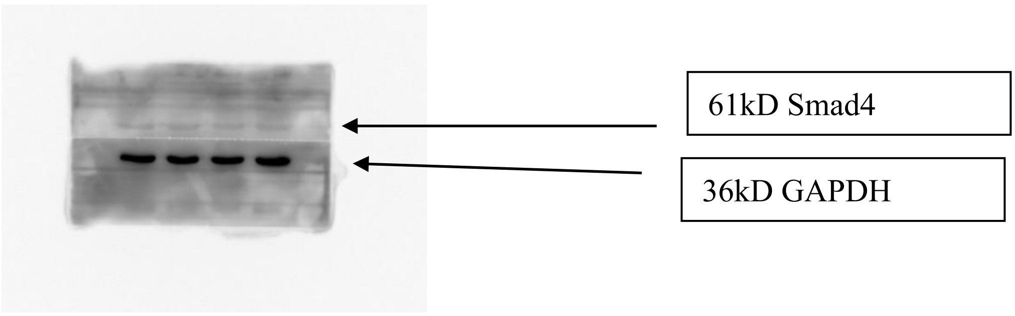

Fig3

3-D

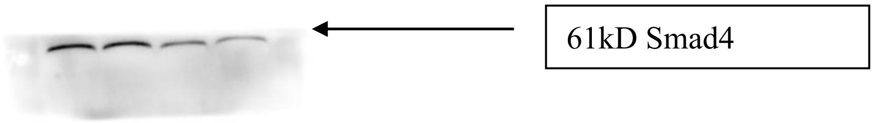

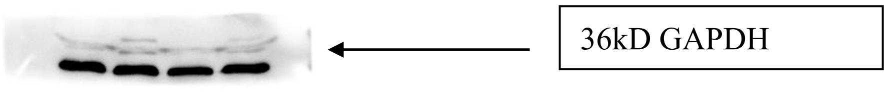

3-G

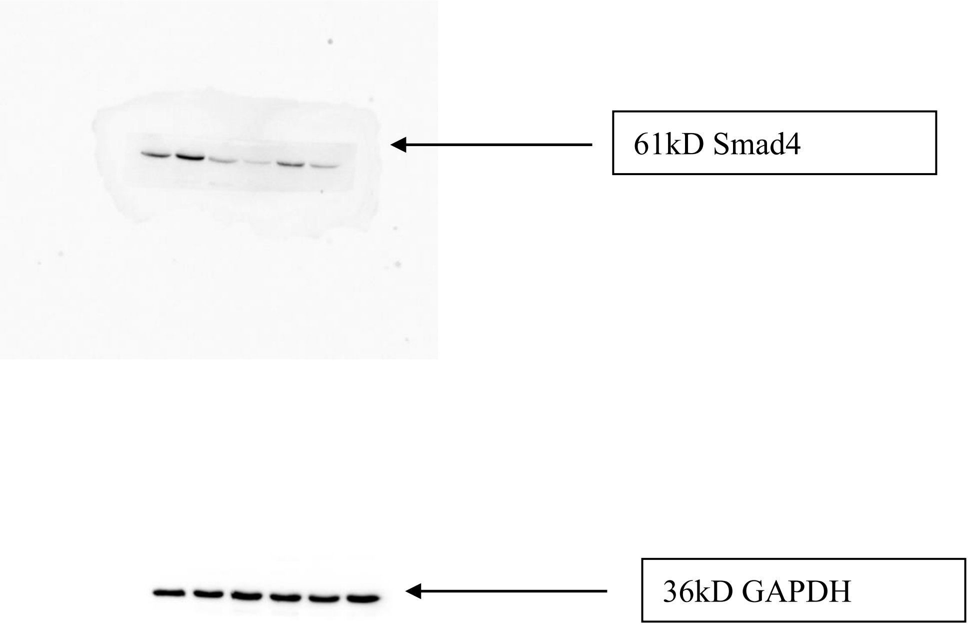

Fig4

4-A

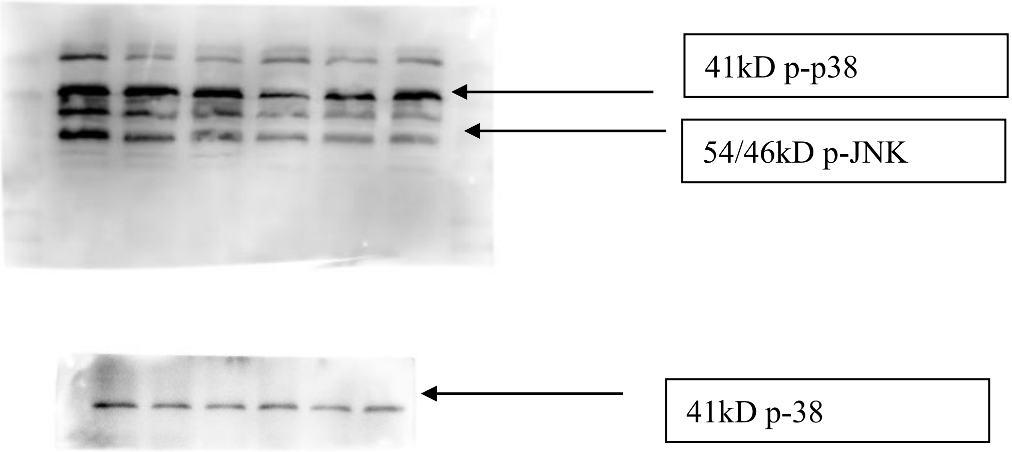

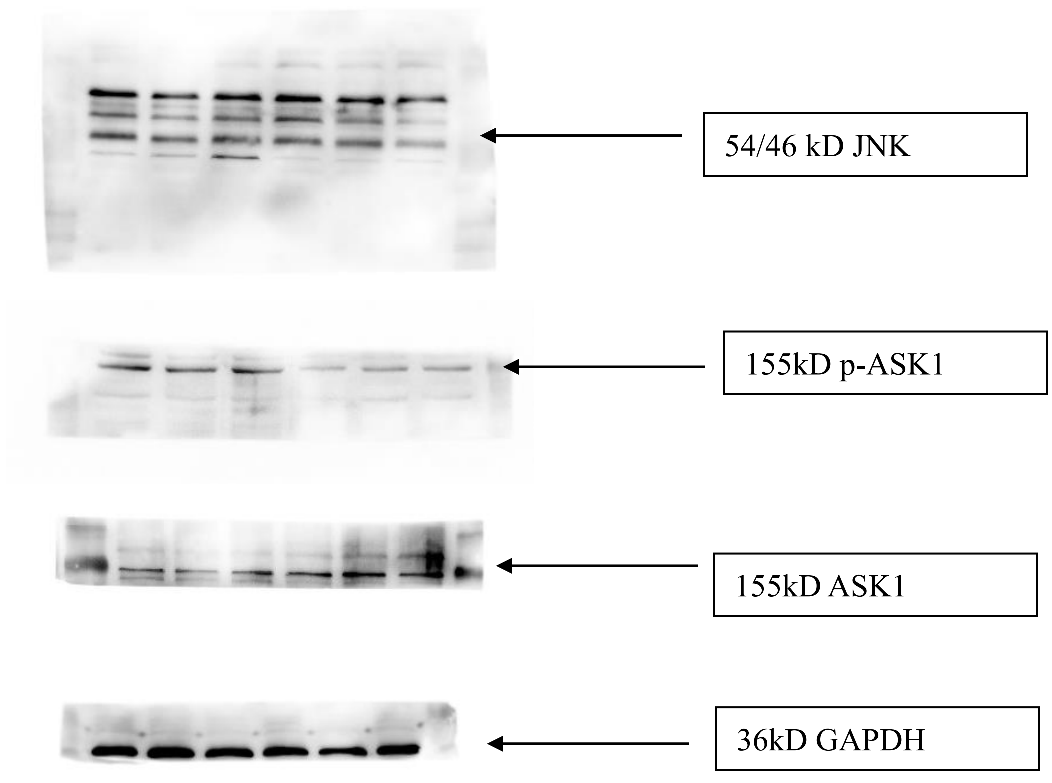
54/46 kD JNK
155kD p-ASK1
155kD ASK1
36kD GAPDH

4-B

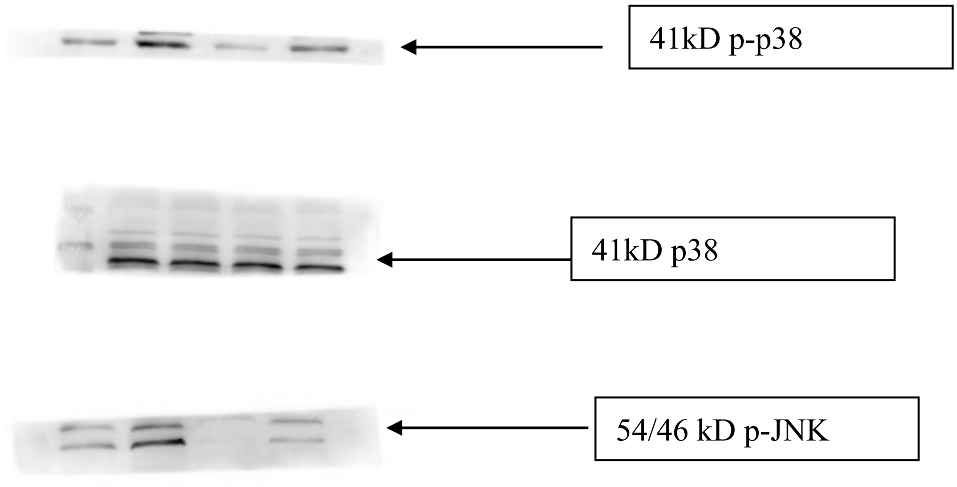
41kD p-p38
41kD p38
54/46 kD p-JNK

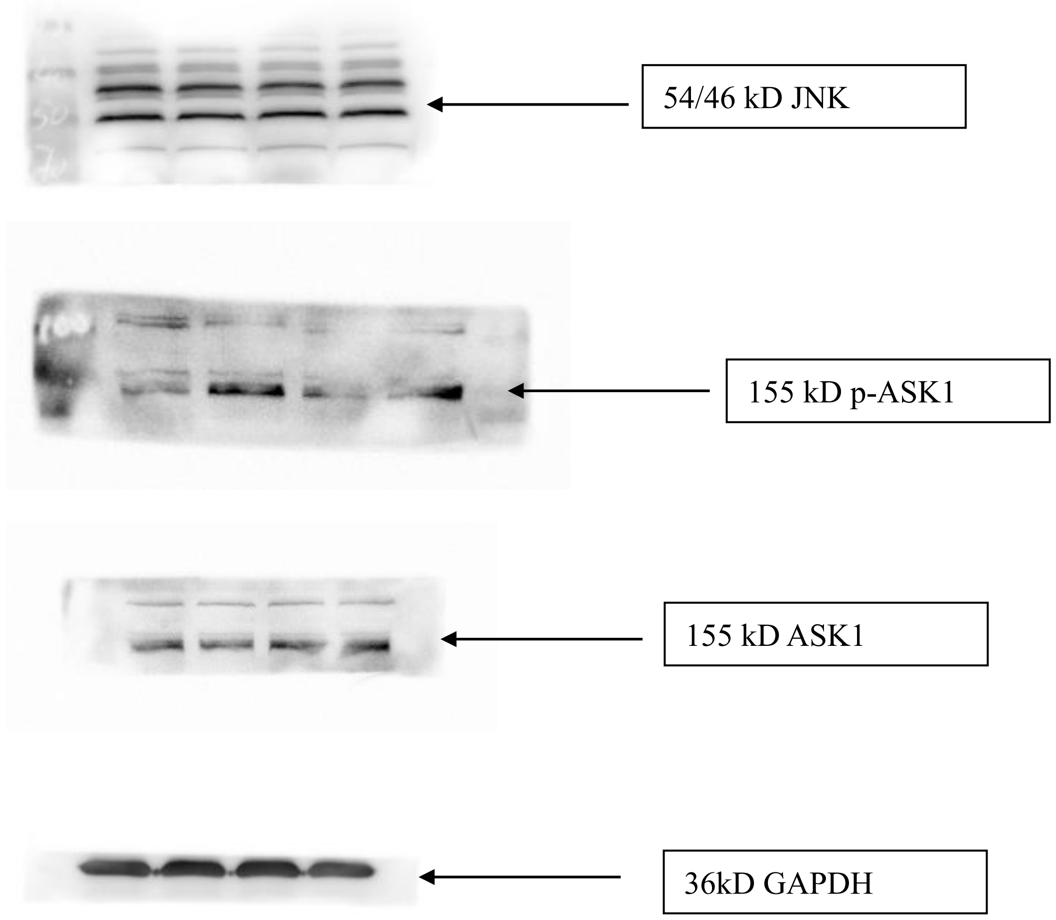
54/46 kD JNK
155 kD p-ASK1
155 kD ASK1
36kD GAPDH

4-C

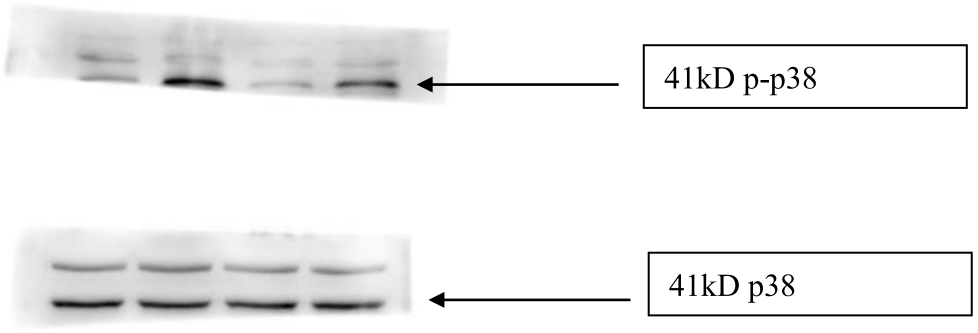
41kD p-p38
41kD p38

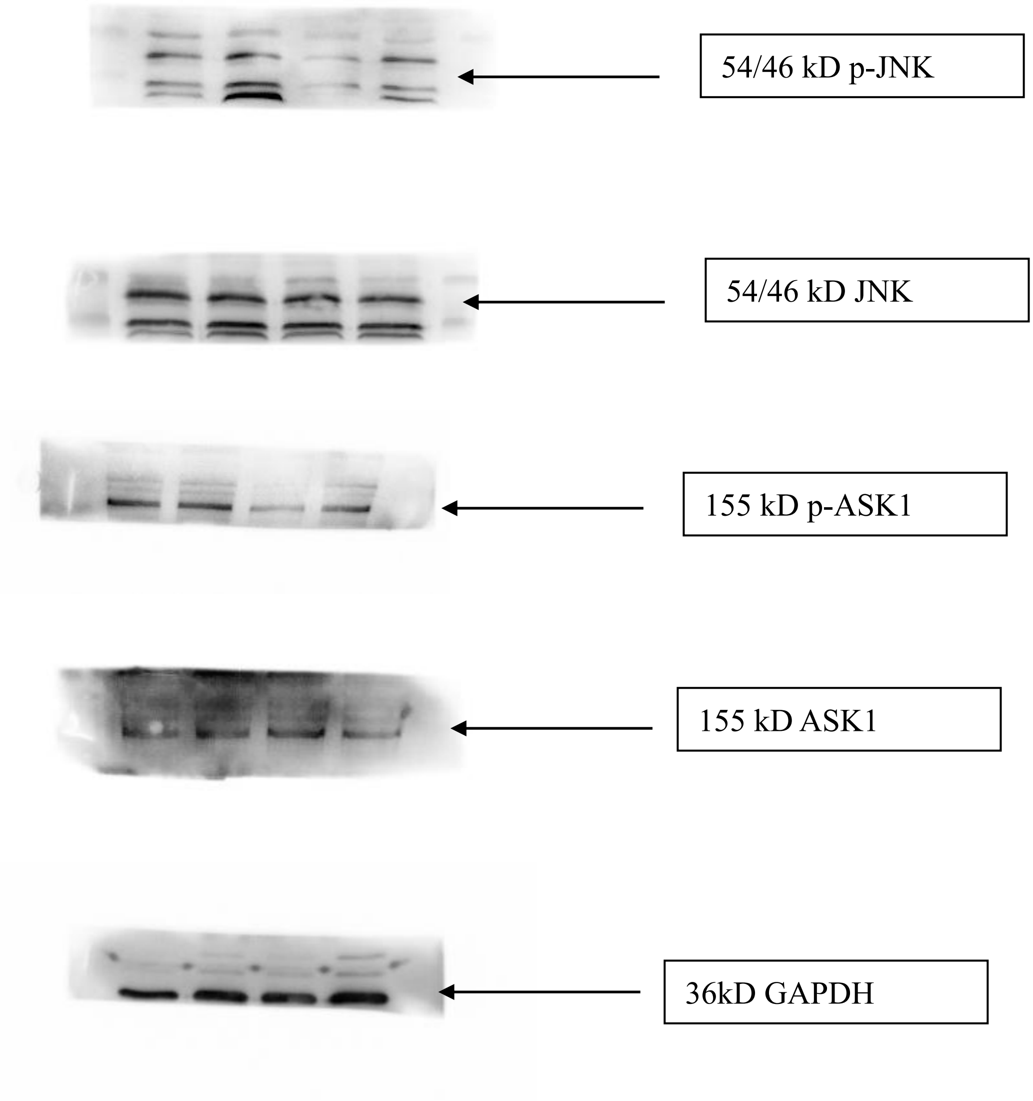
54/46 kD p-JNK
54/46 kD JNK
155 kD p-ASK1
155 kD ASK1
36kD GAPDH

Fig5

5-H

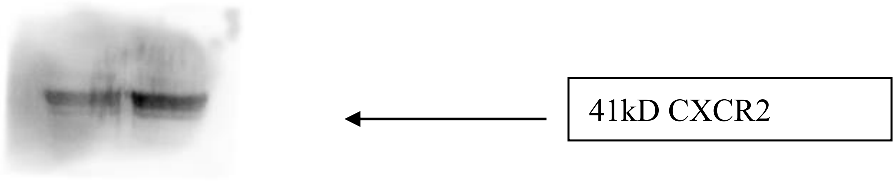
41kD CXCR2

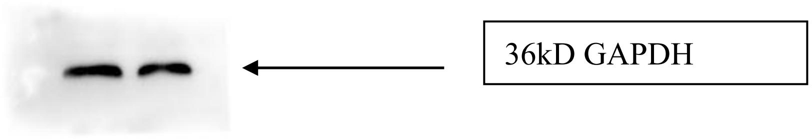

Fig 6

6-E

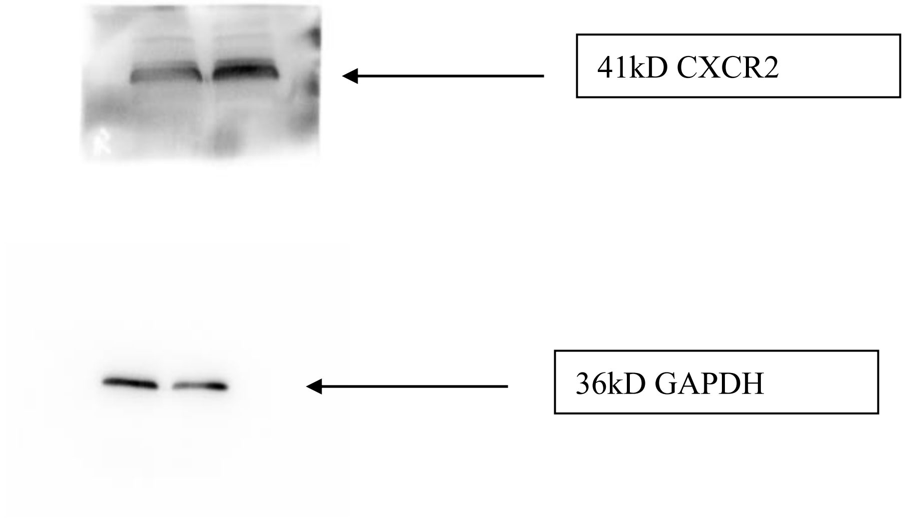

Fig S1

A

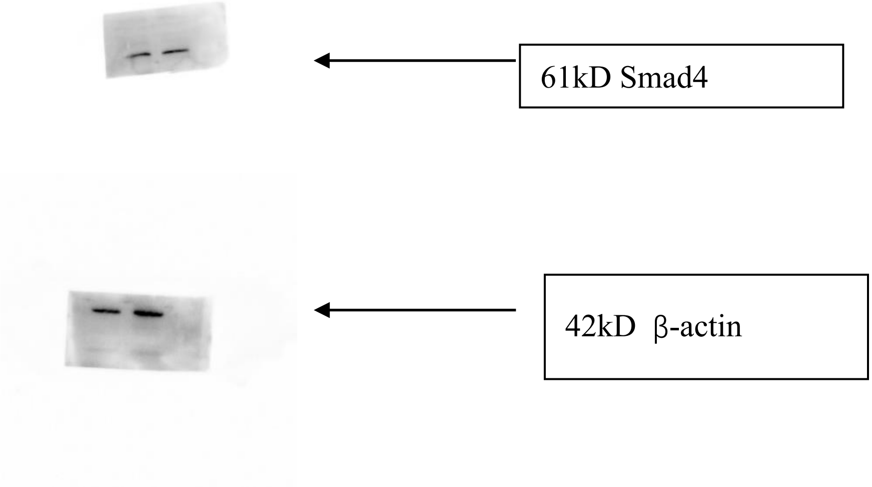

Fig S2

A

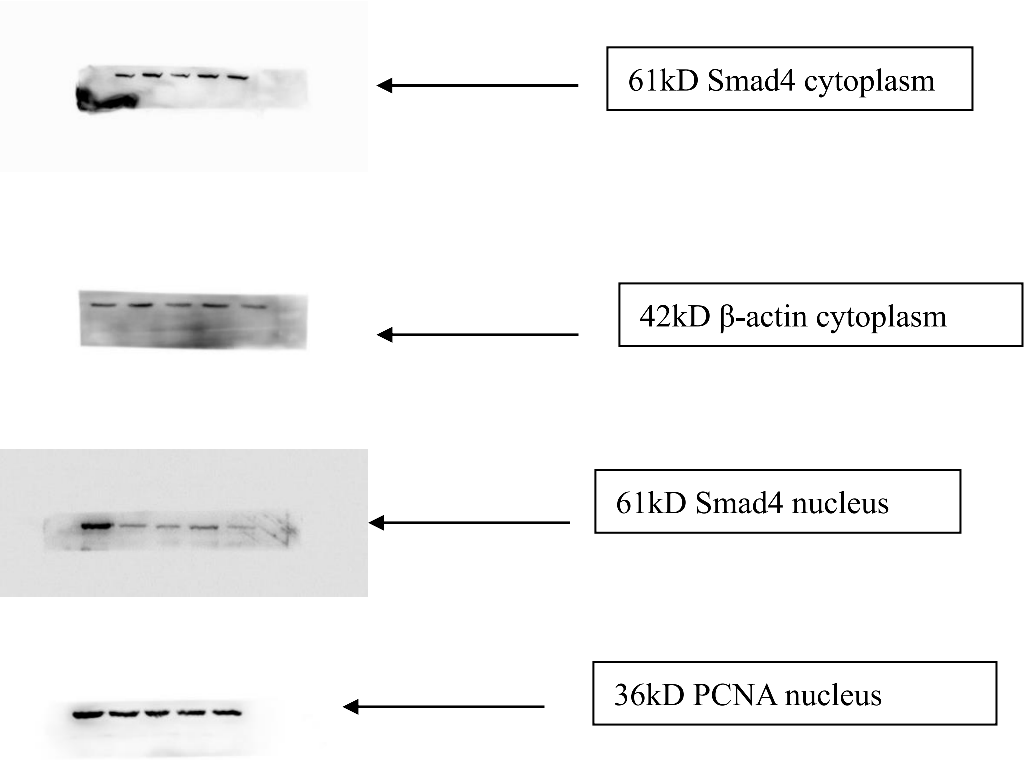

B

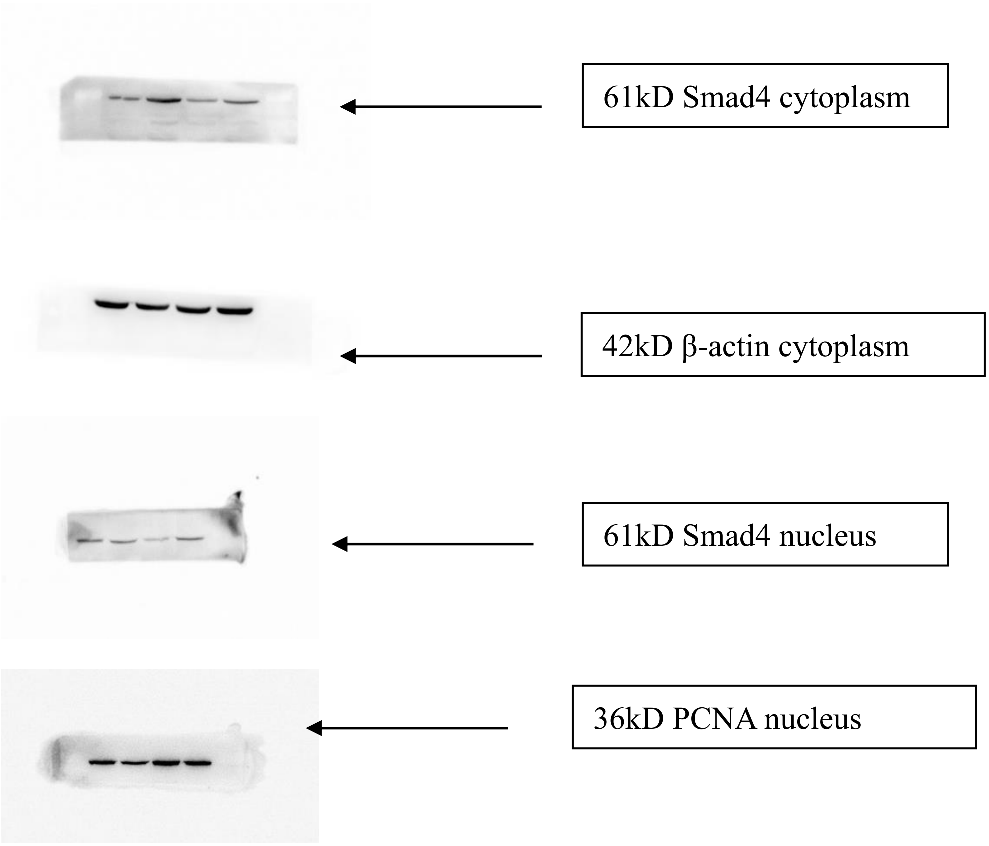
61kD Smad4 cytoplasm
42kD β-actin cytoplasm
61kD Smad4 nucleus
36kD PCNA nucleus
