# Supplementary material for: Smad4 deficiency in hepatocytes attenuates NAFLD progression via inhibition of lipogenesis and macrophage polarization
Source: Cell Death Dis. 2025 Jan 31;16(1):58. doi: 10.1038/s41419-025-07376-8 (PMC11785999; doi:10.1038/s41419-025-07376-8)
Supplement: Supplementary file 3 — Supplemental material [file 41419_2025_7376_MOESM3_ESM.pdf]

Supplementary materials and methods

Tissue microarray immunohistochemistry staining

Tissue microarrays consisting of 5 healthy liver tissues and 10 NASH patient cases for immunohistochemical staining were purchased from Taibosi Biotechnology Company (Xi'an, China). The company provided ethical statements to confirm that the local ethics committees approved their consent procedures. The ethical statement provided by the company and the protocol of the experiment were checked carefully and approved by the Ethics Committee of Beijing Jiaotong University. The tissue microarrays were stained with an anti-Smad4 antibody (ProteinTech, Chicago, IL, USA). Five random fields were observed under a light microscope. The intensity of MyD88 expression was evaluated.

Biochemical assays of blood and liver tissues

Mouse blood samples were centrifuged at 3000 rpm for 8 min to obtain serum. The levels of serum ALT, AST and TC were detected by Vitonglihua Experimental Animal Centre (Beijing, China). Liver tissue was homogenized in cold absolute alcohol and centrifuged at 2500 rpm for 10 minutes to obtain the supernatant. TG and NEFA contents in serum and liver were determined by commercial kits (Nanjing Jiancheng Bioengineering Institute, Jiangsu, China) according to the instructions of manufacturer.

Histology and immunostaining

Paraffin-embedded and frozen liver tissue sections were prepared, then tissue sections were stained with Oil Red O and H&E. For immunofluorescence stain detection, cryostat and frozen sections were incubated with anti-Smad4 antibody (ProteinTech,

Chicago, IL, USA), anti-F4/80, anti-CD11b, and anti-Gr-1 antibodies (BD Pharmingen, San Diego, CA, USA), anti-Albumin, anti-PCNA , anti-CD86, anti-CD206 antibodies (Santa Cruz Biotechnology, Shanghai, China), anti-CXCL1 antibody (Sangon Biotech, Shanghai, China), afterwards incubated with Alexa Fluor 488- or 594-conjugated secondary antibodies (Invitrogen, Carlsbad, CA, USA). For immunohistochemistry (IHC) detection, paraffin-embedded tissue sections were incubated with anti-Albumin and anti-Smad4 antibody, followed by incubation with horseradish peroxidase (HRP)-conjugated secondary antibodies. Images were obtained by using microscope (DP71, OLYMPUS, Tokyo, Japan)

Cellular immunofluorescence and Oil red O stain

The primary hepatocytes and Raw264.7 were plated into 24-well plates (primary hepatocytes: 2.5×10^4 cells/well, Raw264.7: 1×10^5 cells/well). For immunofluorescence staining, cells were fixed in 4% paraformaldehyde for 15 minutes and permeabilized with 0.1% cold triton - x100 for 10 minutes at room temperature. The cells were incubated with 2% BSA for 30 minutes to block nonspecific binding sites. Then, the cells were incubated with the anti- Albumin, anti-Smad4, and anti-CD86 antibodies followed by incubation with Alexa Fluor 488- and Alexa Fluor 594-conjugated secondary antibodies (Invitrogen, Carlsbad, CA, USA). The AML12 cells were plated into 6-well plates (2×10^5 cells/well). Cells were washed with 70% ethanol for a few seconds followed by Oil Red O staining.

Isolation of mouse primary hepatocytes

Primary hepatocytes from 6 to 8 weeks old mice were isolated by using two-step

collagenase perfusion, as described previously. In brief, after anesthesia, mice were perfused with EGTA working solution and collagenase type I solution through the portal vein. Filtered primary hepatocytes were separated through centrifugation at 50 g for 3 min, then purified on 40% Percoll solution and centrifuged at 800 g for 10 min. The primary hepatocytes were seeded on plates which are coated with rat tail collagen type I (CORNING, NY, USA). Mouse primary hepatocytes were cultured in William's E medium (Gibco, Grand Island US) supplemented with 10% FBS and 1% penicillin/streptomycin. To examine the lipid accumulation, primary hepatocytes were treated with 500 μM palmitic acid.

Small interfering RNA (siRNA) interference

AML12 cells were transfected with Smad4-targeting small interfering RNA (si-Smad4) and control siRNA(si-NC) supplemented with siRNA mate transfection reagent (GenePharma, Suzhou, China) according to the protocols of manufacturer.

Cell transfection with lentiviral vector and stable cell line construction

AML12 cells were transfected with Smad4-targeting lentiviral vector(sh-Smad4) and control Lentivirus(sh-GFP) supplemented with HitransG transfection reagent (Genechem, Shanghai, China). Infected AML12 cells were cultured with 2 μg/mL puromycin (InvivoGen, San Diego, CA, USA) to obtain the sh-Smad4 stable cell line.

qRT-PCR

Total RNA in frozen liver tissues and cells was extracted with Trizol reagent (TransGen Biotech, Beijing, China). A total of 2000 ng of RNA was reverse transcribed to cDNA using a Primescript RT Master Mix Kit (MedChemExpress, Princeton, NJ, USA). qRT-

PCR was performed using the SYBR Premix Ex TaqTM Kit (MedChemExpress, Princeton, NJ, USA) according to the manufacturer's protocols. Data were analyzed using the $2^{(-\Delta\Delta Ct)}$ formula and normalized to GAPDH expression.

Western blot analysis

Liver tissues and cultured cells were collected and lysed in RIPA Lysis Buffer (Beyotime, Shanghai, China) supplemented with NaF, PMSF and a cocktail protease inhibitor (Solarbio, Beijing, China). Protein concentrations were determined using a BCA protein assay kit (LABLEAD, Beijing, China). Protein samples were separated by electrophoresis apparatus on a10% SDS-PAGE gel at 115 V for 1.2 h, then were transferred to a PVDF membrane at 200 mA for 1 h. After being blocking with 5% milk in TBST at room temperature for 1h, the membranes were incubated at 4℃ overnight with the following primary antibodies: anti-GAPDH, anti-JNK, anti-p-JNK, anti-p38, and anti-p-p38 (Affinity Biosciences, Cincinnati, OH, USA), anti-Smad4, anti-ASK1 and anti-p-ASK1 (ProteinTech, Chicago, IL, USA), anti-CXCR2 (Bioss, Beijing, China). HRP-conjugated goat anti-rabbit and goat anti-mouse IgG (Solarbio, Beijing, China) were used as secondary antibodies. All specific bands were scanned using a Clinx Science Instrument and quantified with Image J software.

ELISA

AML12 cells were pretreated with p38 inhibitor (SB203580) and JNK inhibitor (SP600125) for 2 hours. After inhibition, cells were co-cultured with inhibitors and PA (500μM) in DMEM/F12 medium. After 24 hours, the supernatant was harvested 24 hours later and centrifuged, then used for subsequent ELISA analysis. The CXCL1

ELISA kit was purchased from CLOUD-CLONE CROP (Wuhan, China). All tests were performed according to the protocols of manufacturer.

Public database analysis

Gene expression data (GSE164760 profiling data) were downloaded as raw signals from the Gene Expression Omnibus (http://www.ncbi.nlm.nih.-gov/geo), analyzed using the Geo2R function from NCBI (https://www.ncbi.nlm.nih.gov/geo/geo2r). The rank sum test was applied to evaluate the differences in the median Smad4 expression levels between patients with NASH and healthy controls.

Supplementary figure S1 S2:

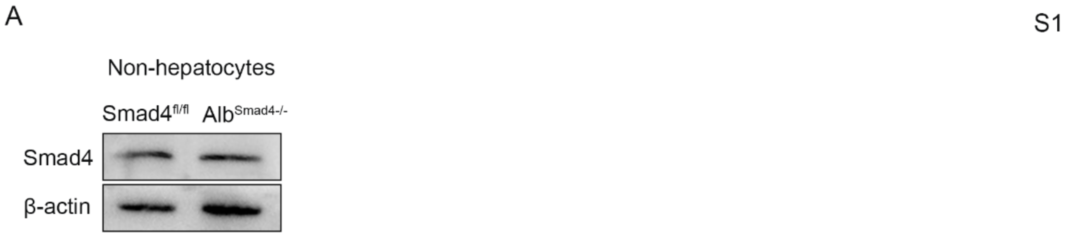

Figure S1: Smad4 was expressed in non-hepatocytes cells of Alb$^{Smad4-/-}$ mice.

(A) Non-hepatocytes were isolated from the liver of Smad4$^{fl/fl}$ and Alb$^{Smad4-/-}$ mice. The protein level of Smad4 in non-hepatocytes was determined using Western blot.

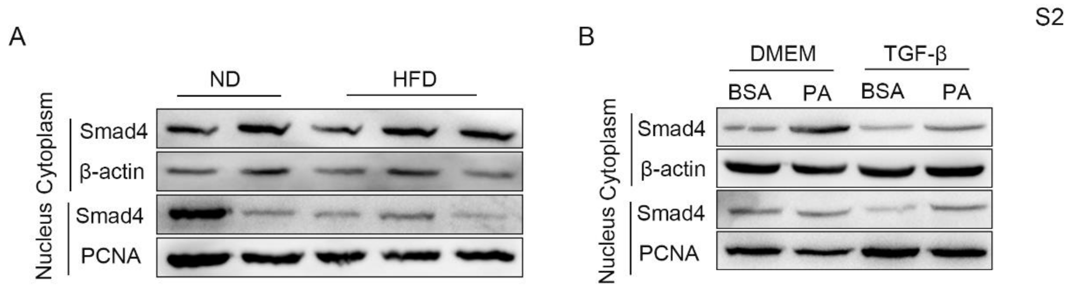

Figure S2: Smad4 activation did not dependent on TGF-β.

(A) The levels of Smad4 in cytoplasm and nucleus in the liver tissues of HFD-treated $Alb^{Smad4-/-}$ and $Smad4^{fl/fl}$ mice were detected by Western blot. (B) AML12 cells were exposed to palmitic acid (PA: 500mM) and gave or not give 10ng/ml TGF-β for 24h. The protein levels of Smad4 in cytoplasm and nucleus were detected by Western blot.
